# Liquid biopsy for detecting epidermal growth factor receptor mutation among patients with non-small cell lung cancer treated with afatinib: a multicenter prospective study

**DOI:** 10.1186/s12885-022-10135-z

**Published:** 2022-10-04

**Authors:** Hiroaki Fujii, Hideyuki Nagakura, Nobuaki Kobayashi, Sousuke Kubo, Katsushi Tanaka, Keisuke Watanabe, Nobuyuki Horita, Yu Hara, Masanori Nishikawa, Kenji Miura, Harumi Koizumi, Yu Ito, Motofumi Tsubakihara, Naoki Miyazawa, Makoto Kudo, Masaharu Shinkai, Takeshi Kaneko

**Affiliations:** 1 Department of Pulmonology, Yokohama City University Graduate School of Medicine, 3-9 Fukuura, Kanazawa-ku, Yokohama, Kanagawa 236-0004 Japan; 2 Department of Internal Medicine, Yokohama Ekisaikai Hospital, 1-2 Yamadacho, Naka-ku, Yokohama, Kanagawa 231-0036 Japan; 3 Respiratory Disease Center, Yokohama City University Medical Center, 4-57 Urafunacho, Minami-ku, Yokohama, Kanagawa Japan; 4 Department of Pulmonology, Fujisawa City Hospital, 2-6-1 Fujisawa, Fujisawashi, Kanagawa 251-8550 Japan; 5 Department of Pulmonology, Yokohama Sakae Kyosai Hospital, 132 Katsuracho, Sakae-ku, Yokohama, Kanagawa 247-8581 Japan; 6 Department of Pulmonology, Yokohama Minami Kyosai Hospital, 1-21-1 Mutsuurahigashi, Kanazawa-ku, Yokohama, Kanagawa 236-0037 Japan; 7 Department of Pulmonology, Yokohama Rosai Hospital, 3211 Kozukicho, Kohoku-ku, , Yokohama, Kanagawa 222-0036 Japan; 8 Department of Pulmonology, National Hospital Organization Yokohama Medical Center, 3-60-2 Harajuku, Totsuka-ku, Yokohama, Kanagawa 245-8575 Japan; 9 Department of Pulmonology, Saiseikai Yokohamashi Nanbu Hospital, 3-2-10 Konandai, Konan-ku, Yokohama, Kanagawa 234-0054 Japan; 10 Department of Respiratory Medicine, Tokyo Shinagawa Hospital, 6-3-22 Higashioi, Shinagawa-ku, Tokyo, 140-8522 Japan

**Keywords:** Cell-free deoxyribonucleic acid, Epidermal growth factor receptor, Liquid biopsy, Non-small cell lung cancer, Second-generation epidermal growth factor receptor (EGFR) tyrosine kinase inhibitors

## Abstract

**Background:**

This study aimed to determine the effectiveness of liquid biopsy in detecting epidermal growth factor receptor (EGFR) mutations at diagnosis, disease progression, and intermediate stages.

**Methods:**

This prospective, multicenter, observational study included 30 patients with non-small cell lung cancer treated with afatinib, harboring a major EGFR mutation confirmed by tumor tissue biopsy. We collected blood samples for liquid biopsy at diagnosis, intermediate stage, and progressive disease. Tissue and liquid biopsies were examined using Cobas ® EGFR Mutation Test v2.

**Results:**

Liquid biopsy detected EGFR mutations in 63.6% of the patients at diagnosis. The presence of metastasis in the extrathoracic, brain, and adrenal glands correlated positively with the detection of EGFR mutations. Patients with positive EGFR mutations at diagnosis had significantly shorter overall and progression-free survival than patients with negative EGFR mutations. Four of the 18 patients (22.2%) who reached progressive disease had positive EGFR T790M mutations. Three of 10 patients (30.0%) with progressive disease were positive and negative for T790M using tumor re-biopsy and liquid biopsy, respectively. The results of EGFR mutation by tissue re-biopsy were the same as those of liquid biopsy in the three patients who were positive for significant EGFR mutations but negative for the T790M mutation using liquid biopsy at progressing disease. Only two patients were positive for major EGFR mutations at intermediate levels.

**Conclusions:**

Liquid biopsy can be a prognostic factor in EGFR-tyrosine kinase inhibitor treatments at diagnosis. Tumor re-biopsy can be omitted in patients with positive EGFR mutations by liquid biopsy at PD.

**Supplementary Information:**

The online version contains supplementary material available at 10.1186/s12885-022-10135-z.

## Background

Non-small cell lung cancer (NSCLC) accounts for approximately 85% of all lung cancers, and adenocarcinoma is the most common histological type. The incidence of epidermal growth factor receptor (EGFR) mutations among Asian patients with adenocarcinoma is approximately 51.4% [1]. Several phase III trials that compared first-line EGFR-tyrosine kinase inhibitors (TKIs) with platinum combination therapy in patients with NSCLC with EGFR mutations have been reported [2–6]. In these trials, the EGFR-TKIs were associated with better progression-free survival (PFS) and overall survival (OS). Therefore, EGFR-TKIs are recommended as the first-line treatment for patients with NSCLC with EGFR mutations. Afatinib, a second-generation EGFR-TKI, is preferred to chemotherapy in treating patients with NSCLC with EGFR mutations [6]. In a phase IIb study (LUX-Lung7), afatinib improved PFS and time-to-treatment failure (TTF) in most patient categories, except light ex-smokers and, for TTF alone, and patients without brain metastases [7]. Therefore, afatinib is considered one of the standard treatments for EGFR mutation-positive advanced NSCLC.

Following treatment with afatinib, approximately 40% of patients developed the T790M mutation in EGFR exon 20 [8]. For these patients, the administration of osimertinib yielded better outcomes than platinum-pemetrexed therapy [1]. Therefore, detecting T790M during therapy with a first or second-generation EGFR-TKI treatment is important.

Traditionally, tumor tissue samples have been utilized for EGFR testing. However, collecting tumor samples from patients is invasive and sometimes unrepeatable. EGFR gene mutations in blood samples were examined using liquid biopsy. It is a non-invasive method that identifies driver oncogene mutations from cell-free DNA (cfDNA) and can be performed repeatedly [9]. Recently, clinical applications of liquid biopsy using cfDNA have been studied by many researchers for early detection of various cancer [10, 11], surveillance of minimal residual diseases, treatment selection for recurrent diseases [12], and treatment response assessment [13].

Therefore, this study aimed to determine if liquid biopsy could substitute tumor biopsy in detecting EGFR mutations and could be used to monitor disease progression in patients on afatinib therapy.

## Methods

### Patients and study design

The inclusion criteria were patients (1) diagnosed with NSCLC, (2) aged ≥ 20 years, (3) who were treatment-naïve, and (4) with a common sensitive EGFR mutation, exon 19 deletion, or L858R mutation in exon 21. Additional inclusion and exclusion criteria are described in Supplementary Tables 1 and 2. All patients signed a written informed consent form. This study followed the guidelines of the Declaration of Helsinki and was approved by the institutional review board at Yokohama City University Hospital (approval number B160804003).

### Collection and analyses of EGFR mutation

Tumor samples were obtained following the diagnosis of NSCLC by biopsy before the commencement of afatinib therapy. Additionally, we obtained tumor samples via re-biopsy at the time of progressive disease (PD). Blood samples for liquid biopsy were collected at pre-treatment, intermediate (8 and 10 months after commencement of afatinib therapy in patients with L858R and Ex19del, respectively), and post-PD. DNA extraction was performed using Cobas ® cfDNA Sample Prep for blood samples or Cobas DNA Sample Prep for tissue samples (Roche Molecular Systems, Inc., Pleasanton, CA, USA). After the quality of the extracted DNA was confirmed to meet the requirements of the following test, EGFR testing was performed using Cobas ® EGFR Mutation Test v2 (Roche Molecular Systems, Inc., Pleasanton, CA, USA). The EGFR testing in this study complied with the manufacturer's protocol, and the validation assay was omitted.

### Treatments

Patients were administered 40 mg of afatinib each day until disease progression or intolerable toxicity occurred. Treatment with afatinib can be continued even in patients with PD because treatment interruptions and dose reductions can prevent and manage adverse events (AEs). Despite the best supportive care, dose modifications made for Common Terminology Criteria for Adverse Events grade 3 or persistent grade 2 AEs, we discontinued afatinib until the severity of AEs returned to grade 1 or baseline. Afatinib was re-commenced at a reduced dose of 10 mg increments to a maximum daily dose of 20 mg; otherwise, dosing was permanently terminated at the discretion of the attending doctor.

### Outcomes

The primary endpoint was the incidence of EGFR T790M mutation expression in the liquid biopsy of cfDNA. Subsequently, the incidences at pre-treatment, intermediate, and PD of the liquid biopsy were compared with those of tissue biopsy. The secondary outcome was the efficacy and safety of afatinib therapy. PFS was defined as survival without disease progression or death and was calculated as the time from the administration of afatinib until the first observation of disease progression. OS was defined as the time from the initiation of afatinib treatment until death or the last follow-up visit. AEs were recorded by the investigator at pre-treatment and each visit according to the National Cancer Institute Common Terminology Criteria for Adverse Events (version 4.0).

### Statistical analysis

Associations between clinical characteristics and the treatment response to afatinib were analyzed using Fisher's exact test or the chi-square test. Survival analysis was performed using Kaplan–Meier estimation to assess differences between the groups. Statistical significance was set at *P* ≤ 0.05. All statistical analyses were performed using JMP Pro 15.0 (SAS Institute Inc.).

## Results

### Patient characteristics

This prospective observational study enrolled 30 patients between August 2016 and April 2021. Baseline characteristics and safety data were analyzed in an intention-to-treat (ITT) population. The patient characteristics are summarized in Table 1. The median age of the patients was 69.0 years (range 54–82). Pathologically, 28 (93.3%) patients had adenocarcinoma, and two (6.6%) had adenosquamous carcinoma. Regarding performance status, 22 (73.3%), six (20.0%), and two (6.6%) patients had Eastern Cooperative Oncology Group performance status scores of 0, 1, and 2, respectively. Regarding clinical stages, two patients were stage IIIB, 21 patients were stage IV, and seven patients had postoperative recurrence. Regarding EGFR gene mutations, 25 (83.3%) patients had a 19del mutation, and five (16.6%) had an L858R mutation. At the time of enrollment, EGFR mutations in the blood were detected in 19 patients (63.3%).

**Table 1:** Patient characteristics

Enrolled patients, n	30
Male sex, n (%)	7 (23.3)
Age, Median (range)	69.0 (54–82)
Performance status, n (%)	
0	22 (73.3)
1	6 (20.0)
2	2 (6.6)
3	0
4	0
Smoking history (pack-years)	
0	16
1–19	5
≥ 20	9
Histology, n (%)	
adenocarcinoma	28 (93.3)
adenosquamous	2 (6.6)
Stage, n (%)	
IIIB	2 (6.6)
IV	21 (70.0)
postoperative recurrence	7 (23.3)
Type of mutations, n (%)	
19del	25 (83.3)
L858R	5 (16.6)
Metastasis, n (%)	
brain	7 (23.3)
liver	4 (13.3)
adrenal	7 (23.3)
bone	16 (53.3)
malignant pleural effusion	10 (33.3)
extrathoracic	20 (66.6)

### Clinical course of enrolled patients

**Fig. 1:**
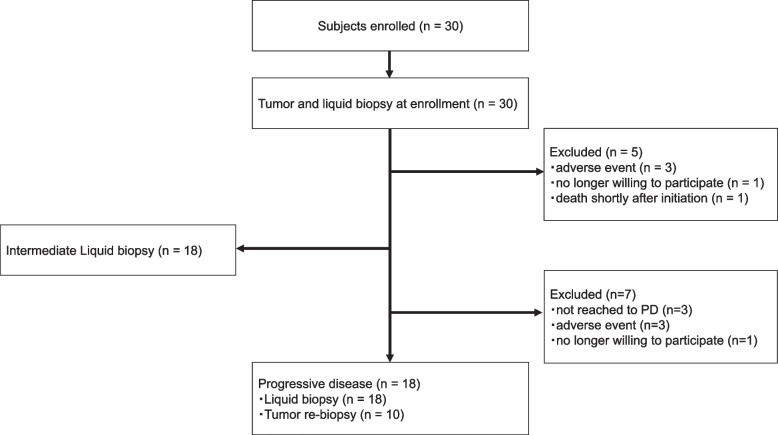
Flowchart showing the process used for study selection. Thirty patients were enrolled, all with tumor and liquid biopsies at enrollment. A total of five patients were excluded: three had adverse events, one was unwilling to participate, and one died shortly after starting. Eighteen patients underwent an interim liquid biopsy. After the interim liquid biopsy, seven patients were also excluded: three did not reach PD, three had adverse events, and one was unwilling to participate. Finally, 18 patients reached PD, of whom 18 had liquid biopsy and 10 had tumor re-biopsy. PD, progressive disease

Three of the 30 patients were still on afatinib therapy during the final analysis. The most frequent reason for treatment termination was disease progression (18 patients), followed by AEs (six patients) and patient choice (two patients) (Fig. 1). The overall response rate (ORR) was assessed in patients who received at least one treatment and for whom a response assessment was conducted. PFS and OS were analyzed in the ITT population.

### Results and comparison of EGFR testing between tumor and blood biopsy

At diagnosis, all 30 patients were examined using liquid biopsy. Blood samples from 19 patients had the same EGFR mutation as those in the tumor samples (63.3%, Supplementary Table 3). Four of 18 patients who reached PD were positive for the EGFR T790M mutation using liquid biopsy (22.2%, Table 2). Three out of 10 patients examined with tumor and liquid biopsies were positive and negative for T790M, respectively (30.0%, Table 2). In contrast, four patients who were positive for T790M by liquid biopsy were not eligible for tumor biopsy. Among the 10 patients who underwent both tumor and liquid biopsy at PD, the same EGFR mutations were detected in four patients by both methods (40.0%, Table 2). The results of EGFR mutation by tumor tissue re-biopsy were the same as those of liquid biopsy in the three patients who were positive for major EGFR mutations and negative for the T790M mutation by liquid biopsy at PD (100%) (Supplementary Table 4). Two patients out of 18 were positive for the 19del mutation (11.1%, Supplementary Table 5) at the intermediate and pre-treatment stage using liquid biopsy. All patients were negative for the T790M mutation at the intermediate stage.

**Table 2:** ^**†**^EGFR mutation for each patient who underwent tumor re-biopsy and liquid biopsy at ^‡^PD time

EGFR mutation	Tumor (*n* = 10)	Liquid (*n* = 18)
19del/ T790M	2 (20.0%)	4 (22.2%)
L858R/ T790M	1 (10.0%)	0
19del/ -	4 (40.0%)	3 (16.7%)
L858R/ -	1 (10.0%)	1 (5.6%)
negative	1 (10.0%)	10 (55.6%)
unsuccessful	1 (10.0%)	0
concordance between tumor and liquid biopsy	4/10 (40.0%)	

^†^*EGFR* Epidermal growth factor receptor, ^‡^*PD* Progressive disease

### Factors related to the detection of EGFR mutation by liquid biopsy

To identify the factors related to the detection of EGFR mutations in blood samples, clinical variables were compared between the positive and negative results in EGFR testing by liquid biopsy (Table 3). There were significant differences in alkaline phosphatase (ALP) and extra-thoracic, brain, and adrenal metastases between the positive and negative results in EGFR testing from blood samples (ALP: 304 U/L vs. 225 U/L, *P* = 0.0048; extrathoracic metastasis: 89.4% vs. 27.2%, *P* = 0.0010; brain metastases: 36.8% vs. 0%, *P* = 0.0292; adrenal metastases: 36.8% vs. 0%, *P* = 0.0292, log-rank).

**Table 3:** Factors related to detection of ^†^EGFR mutation by liquid biopsy

	EGFR mutation positive (*n* = 19)	EGFR mutation negative (*n* = 11)	*P* value
Age, median (range), years	68.0 (54–82)	69.0 (55–81)	0.5315
histology, n (%) (adenocarcinoma/ adenosquamous)	19 (89.4%) (17/2)	11 (100%) (11/0)	0.5195
N2 or higher, n (%)	13 (68.4%)	5 (45.4%)	0.2663
M factor, n (%)	18 (89.4%)	9 (81.8%)	0.5367
LDH, median, U/L	217	189	0.0776
ALP, median, U/L	304	225	0.0048
CRP, median, mg/dl	0.53	0.12	0.0501
CEA, median, ng/mL	39.1	10.6	0.2307
extrathoracic metastasis, n (%)	17 (89.4%)	3 (27.2%)	0.0010
brain metastasis, n (%)	7 (36.8%)	0	0.0292
liver metastasis, n (%)	5 (26.3%)	0	0.0527
adrenal metastasis, n (%)	7 (36.8%)	0	0.0292
bone metastasis, n (%)	12 (63.1%)	3 (27.2%)	0.1281

^†^*EGFR* Epidermal growth factor receptor, *LDH* Lactate dehydrogenase, *ALP* Alkaline phosphatase, *CRP* C-reactive protein, *CEA* carcinoembryonic antigen

### Treatment efficacy and toxicity of afatinib

**Fig. 2:**
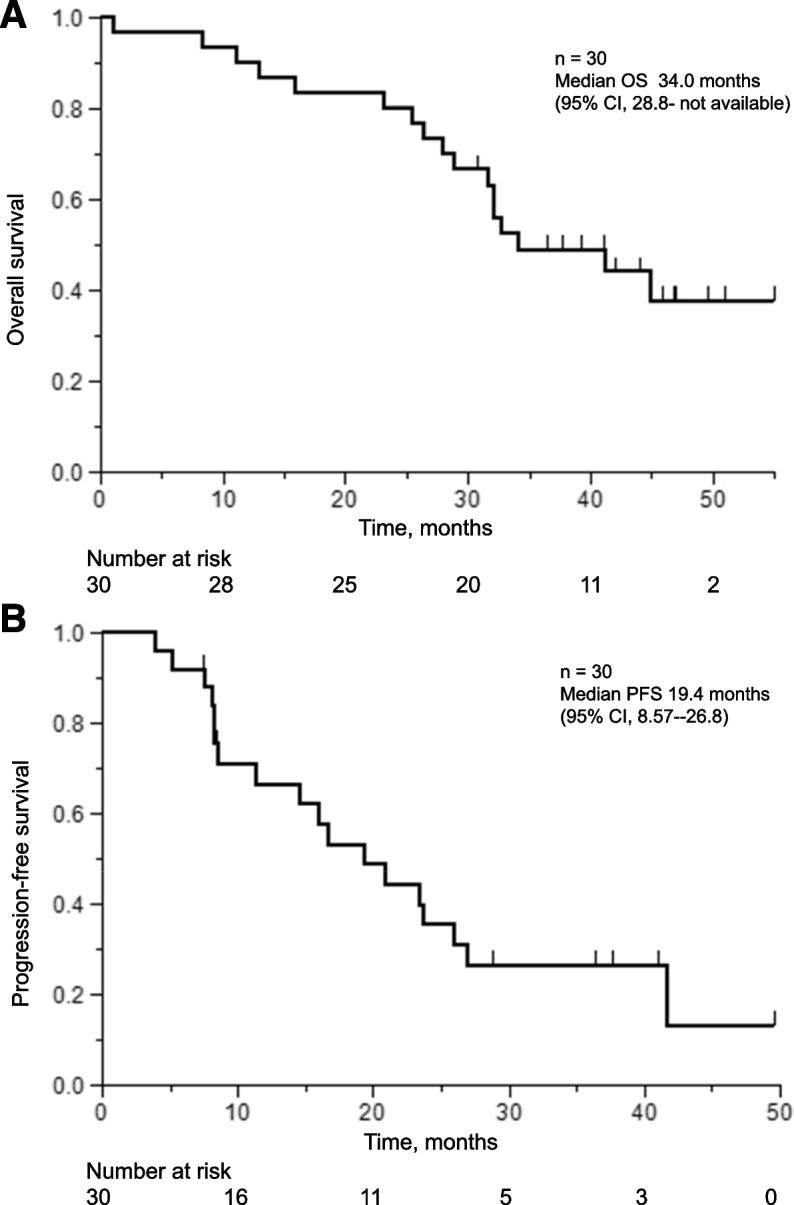
**a** Kaplan–Meier curves of OS. For the analysis of OS, data for any patients who were dead at the time of the analysis were censored at the last recorded date on which the patient was known to be alive. Median OS: 34.0 months (95% CI, 28.8–unavailable). **b** Kaplan–Meier curves of PFS. Median PFS: 19.4 months (95% CI, 8.57–26.8). CI, confidence interval; HR, hazard ratio; OS, overall survival; PFS, progression-free survival

PFS and OS were analyzed in the ITT population. Median OS and PFS were 34.0 months (95% confidence interval [CI], 28.8 months–not reached) and 19.4 months (95% CI, 8.57–26.8 months), respectively (Fig. 2a, b). The median observation time was 34.0 months. Seventeen (56.6%) patients died, and 18 (60.0%) reached PD after afatinib treatment. The ORR was 53.3%. The AEs associated with afatinib are presented in Supplementary Tables 6 and 7. The AEs observed in this study were similar to those reported previously. Grade 3 AEs occurred in 10 patients (33.3%). No grade 4 or treatment-related deaths were observed. Afatinib was terminated in six patients (20%) due to AEs.

### Liquid biopsy as a potential prognostic biomarker

**Fig. 3:**
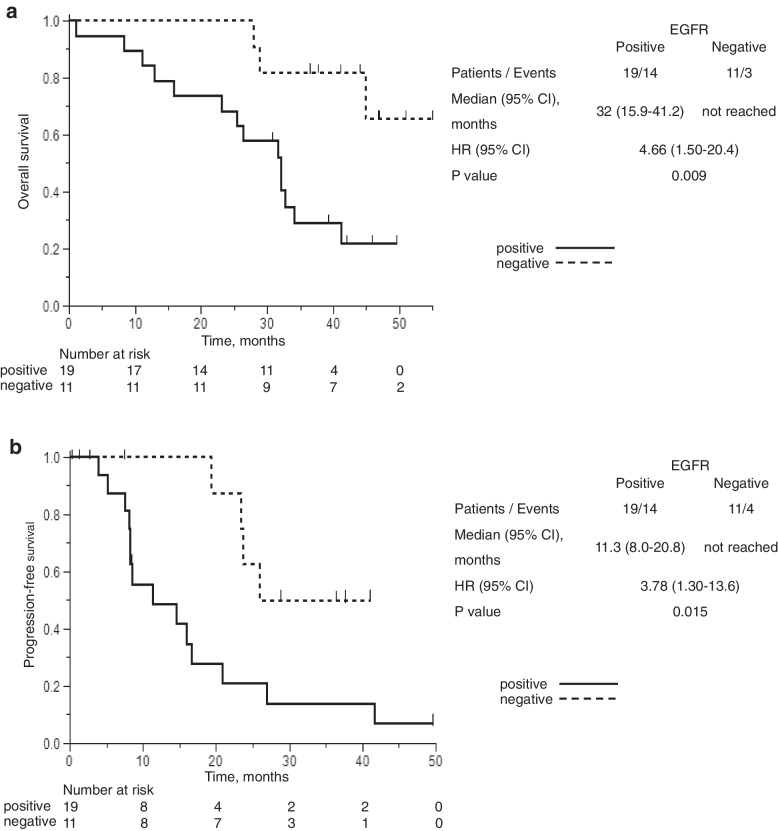
**a** Kaplan–Meier curves of OS divided by positive and negative EGFR mutations by liquid biopsy at diagnosis. Median OS among patients positive for EGFR mutation from blood at pre-treatment was significantly shorter than that of negative patients (32 months vs not reached, HR, 4.66; 95% CI 1.50–20.4; *P* = 0.009) (**b**) Kaplan–Meier curves of PFS divided by positive and negative EGFR mutations by liquid biopsy at diagnosis. PFS among patients positive for EGFR mutation from blood at pre-treatment was significantly shorter than that of negative patients (11.3 months vs not reached, HR, 3.78; 95% CI 1.30–13.6; *P* = 0.015). CI, confidence interval; HR, hazard ratio; OS, overall survival; PFS, progression-free survival

OS and PFS were compared among patients based on the results of EGFR testing using liquid biopsy at the pre-treatment stage (Figs. 3a and b). OS among patients positive for EGFR mutation from liquid biopsy at pre-treatment was significantly shorter than that of negative patients (32 months vs. not reached, hazard ratio [HR], 4.66; 95% CI 1.50–20.4; *P* = 0.009). PFS among positive patients was also significantly shorter than that of negative patients (11.3 months vs not reached, HR, 3.78; 95% CI 1.30–13.6; *P* = 0.015).

**Fig. 4:**
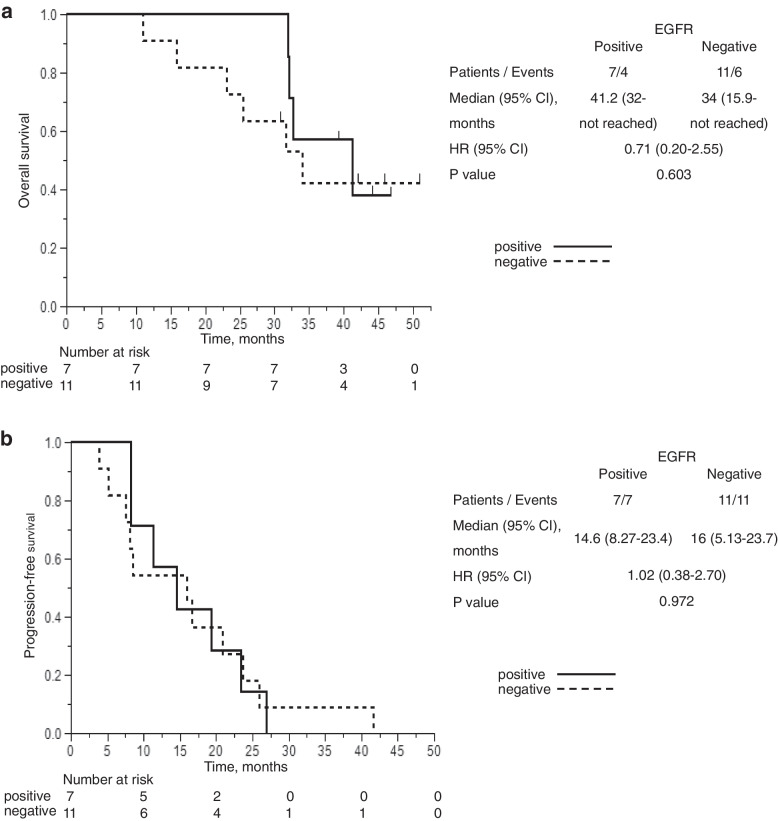
**a** Kaplan–Meier curve of OS in patients expressing the T790M resistance gene at PD. The median OS of patients positive for T790M was not significantly different compared to negative patients (41.2 vs. 34 months, HR, 0.71; 95% CI 0.20–2.55; *P* = 0.603). **b** Kaplan–Meier curve of PFS in patients expressing the T790M resistance gene at PD. The median PFS of patients positive for T790M was not significantly different compared to negative patients (14.6 vs. 16 months, HR, 1.02; 95% CI 0.38–2.70; *P* = 0.972). CI, confidence interval; HR, hazard ratio; OS, overall survival; PFS, progression-free survival; PD, progressive disease

OS and PFS were compared in patients with and without the T790M mutation at PD (Fig. 4a, b). There were no significant differences between the two groups. However, the ORR of T790M positive patients was higher than that of negative patients without a significant difference (85.7% positive, 54.5% negative, *P* = 0.315, Supplementary Table 4).

Eleven patients received osimertinib as post-treatment for afatinib, which was discontinued due to its AEs. Four underwent liquid biopsy at PD, three had tissue re-biopsy at PD, and were confirmed T790M-positive, indicating a switch to osimertinib. One patient was negative for T790M at PD but continued on afatinib after PD, then switched to osimertinib because another liquid biopsy was positive for T790M. Two patients discontinued afatinib and switched to osimertinib because of AEs. One patient changed from afatinib to gefitinib because of AEs, and then to osimertinib because both tissue re-biopsy and liquid biopsy were positive for the T790M mutation.

## Discussion

This prospective, multicenter, observational trial was conducted to elucidate the efficacy of liquid biopsy in detecting EGFR mutations in patients with NSCLC on afatinib therapy in Japan. At diagnosis, liquid biopsy could detect 19 cases (63.3%) among patients with EGFR mutations, as proven by tumor biopsy (Supplementary Table 3). EGFR T790M mutations were detected in four (22.2%) patients who reached PD and were examined by liquid biopsy (Table 2). The OS and PFS of patients with detectable EGFR mutations by liquid biopsy were significantly shorter than those with undetectable EGFR mutations (Fig. 3a, b).

Molecular diagnosis is necessary for the clinical management of patients with NSCLC because molecular targeting agents are more efficient in cases harboring driver oncogene mutations. Traditionally, gene mutations have been tested in tumor tissue samples. Liquid biopsy, which involves gene testing using blood samples, is becoming more popular because it is less invasive and useful in cases of insufficient tumor tissue. The Food and Drug Administration has already approved polymerase chain reaction (PCR)- and next-generation gene-sequencing (NGS)-based methods for liquid biopsy to detect driver oncogene mutations [14].

Some guidelines from the National Comprehensive Cancer Network and the European Society for Medical Oncology also recommend liquid biopsy for the detection of EGFR mutations [15, 16]. In contrast, the Japanese Lung Cancer Society states that plasma testing should only be performed when it is difficult to perform EGFR gene testing on lung cancer tissue specimens for medical reasons [17].

The sensitivity of liquid biopsy using the Cobas ® EGFR Mutation Test v2 has been reported to be 37.9–74.0% [18–20]. Liquid biopsy sensitivity at diagnosis in our study was within this range (63.3%, Supplementary Table 3). As shown in Table 3, liquid biopsy sensitivity for detecting EGFR mutations was higher in cases with distant metastases. In a phase III trial of afatinib (LUX-lung 3 and 6), EGFR mutation detection using liquid biopsy was significantly related to the number of metastases [21]. Therefore, with proper patient selection, liquid biopsy sensitivity can be improved because metastasis seems to reflect the amount of DNA shed into the bloodstream. Additionally, more sensitive detection methods, such as droplet digital PCR (ddPCR) and NGS, may increase the sensitivity of liquid biopsies. The reported sensitivity of ddPCR was 93.5–100% [22, 23].

The detection of the T790M resistant gene mutation is important for patients treated with first- or second-generation EGFR-TKIs. In our study, T790M was detected by liquid biopsy in four of the 18 patients who reached PD (22.2%, Table 2). Others have reported various sensitivities of T790M detection in a blood sample using a Cobas ® detection kit. Koyama, et al. reported that the success rate of liquid biopsy was 43.8% [24]. In the JP-CLEAR trial, the sensitivity of plasma T790M using the cobas® EGFR Mutation Test was 21.1% [25]. Based on the data from tissue biopsy, we previously reported that the incidence of T790M at PD among patients treated with afatinib was 40.2%, which was lower than those of first-generation EGFR-TKIs (52.5%) [8].

Liquid biopsy can provide the opportunity to search for driver oncogene mutations, even in patients with insufficient tumor samples; hence, the combination of liquid biopsy with tumor biopsy increases the detection rate of the T790M resistance gene.

Liquid biopsy also provides an opportunity to track disease progression throughout treatment or predict recurrence following adjuvant therapy [26–29]. We identified a significant relationship between positive EGFR mutation in liquid biopsy and the existence of metastasis (Table 3); hence, positivity in liquid biopsy has been linked to more advanced diseases, including metastasis and poorer performance status [17, 30]. Therefore, it is understandable that positive EGFR mutation by liquid biopsy at diagnosis was identified as a poor prognostic factor, as shown in Figs. 3a and b.

We performed intermediate liquid biopsy 10 and 8 months after the initiation of afatinib in cases of Ex19del and L858R mutations, respectively. In this setting, only two patients were positive for the original EGFR mutation. Other groups reported that molecular progression, indicated by detectable EGFR in plasma, was detected 1.5–2.2 months before clinical progression [16, 31]. Our intermediate liquid biopsy might be too early to detect molecular progression because PFS after afatinib in this study was 19.4 months (Fig. 2b). The clearance of cfDNA and circulating tumor DNA (ctDNA) are promising to evaluate the treatment efficacy of anti-cancer therapy among patients with NSCLC. According to Song et al., patients having driver mutation clearance and ctDNA clearance at any course of chemotherapy were related to higher PFS and OS [32]. However, in our study, there were no statistically significant differences in OS (HR, 1.50; 95% CI 0.43–5.22 *P* = 0.517) and PFS (HR, 0.69; 95% CI 0.22–2.15; *P* = 0.521) between patients with and without clearance of EGFR mutation. This may be due to our study’s limited number of cases. CtDNA kinetics have also been reported effective for the early detection of molecular PD. Shenglin et al. revealed that molecular PD, indicated by the emergence of new mutations or an increase in pre-existing mutations, was identified with a mean lead time of 2.5 months before radiological PD in their longitudinal ctCNA trial [33].

This study had several limitations. First, the sample size was small. Second, the sensitivity of the Cobas ® EGFR Mutation Test v2 kit might be insufficient. For the minimum detection sensitivity of the plasma test using this method, the detection limit of the mutant DNA was 100 copies/ml in 100,000 copies/mL of wild-type DNA. Conversely, ddPCR, used in Maximilian J Hochmair's study, identified T790M in less than 10 copies/ml with a minimum detection sensitivity of 0.01%. A higher T790M positivity rate (73%) after afatinib was reported using ddPCR in their study [34]. Every patient was not examined by both tumor and liquid biopsy at PD because some patients had no suitable lesion for tumor tissue biopsy following treatment with afatinib. Future studies should include a large number of patients, require the collection of both tissue and plasma samples, and use highly sensitive and quantitative methods such as ddPCR and NGS.

## Conclusions

A liquid biopsy at diagnosis can be a prognostic factor for EGFR-TKI treatment. Tumor re-biopsy can be omitted in patients with positive EGFR mutations by liquid biopsy during PD.

## Supplementary Information

**Additional file 1: Supplementary Table 1. **Inclusion criteria. **Supplementary Table 2. **Exclusion criteria. **Supplementary Table 3. **EGFR mutations found in pre-treatment biopsies. **Supplementary Table 4. **Results of ^†^EGFR testing among 18 patients who reached ^‡^PD. **Supplementary Table 5.** ^†^EGFR mutation found by liquid biopsy at intermediate. **Supplementary Table 6. **Safety summary in patients treated with afatinib. **Supplementary Table 7. **All-cause adverse events.

## Data Availability

The datasets used and/or analyzed during the current study are available from the corresponding author upon reasonable request.

## Abbreviations

ALP: Alkaline phosphatase
ALT: Aspartate aminotransferase
ALT: Alanine aminotransferase
Aes: Adverse event
cfDNA: Cell-free deoxyribonucleic acid
CI: Confidence interval
CR: Complete response
ddPCR: Droplet digital polymerase chain reaction
EGFR: Epidermal growth factor receptor
ECOG PS: Eastern Cooperative Oncology Group performance status
HR: Hazard ratio
ITT: Intention-to-treat
N/A: Not available
NGS: Next-generation gene-sequencing
NSCLC: Non-small cell lung cancer
ORR: Overall response rate
OS: Overall survival
PaO_2_: Partial pressure of oxygen
PD: Progressive disease
PFS: Progression-free survival
PR: Partial response
SD: Stable disease
TKIs: Tyrosine kinase inhibitors
TTF: Time-to-treatment failure
