# Supplementary material for: Liquid biopsy for detecting epidermal growth factor receptor mutation among patients with non-small cell lung cancer treated with afatinib: a multicenter prospective study
Source: BMC Cancer. 2022 Oct 4;22:1035. doi: 10.1186/s12885-022-10135-z (PMC9531433; doi:10.1186/s12885-022-10135-z)
Supplement: Supplementary file 1 — Additional file 1: Supplementary Table 1. Inclusion criteria. Supplementary Table 2. Exclusion criteria. Supplementary Table 3. EGFR mutations found in pre-treatment biopsies. Supplementary Table 4. Results of †EGFR testing among 18 patients who reached ‡PD. Supplementary Table 5. †EGFR mutation found by liquid biopsy at intermediate. Supplementary Table 6. Safety summary in patients treated with afatinib. Supplementary Table 7. All-cause adverse events. [file 12885_2022_10135_MOESM1_ESM.docx]

**Supplementary Table 1.** Inclusion criteria

| Inclusion criteria |
| --- |
| 1) Capable of collecting 10 mL new plasma samples 3 times |
| 2) Histologically or cytologically confirmed non-small cell lung cancer other than squamous cell carcinoma |
| 3) Clinical stage IIIB, stage IV, or postoperative recurrence |
| 4) Susceptible ^†^EGFR mutation (deletion mutation in Exon19 or L858R mutation in Exon21) |
| 5) ^‡^EGFR-TKI untreated and scheduled for EGFR-TKI treatment |
| 6) Patients previously treated with radiotherapy were included if all of the following conditions were met  - No radiation irradiation to the evaluation lesion  - Irradiation to bone lesions in the thorax more than 12 weeks after the last irradiation date  - Irradiation to regions other than the thoracic region more than 2 weeks after the last irradiation date |
| 7) The following periods have elapsed since the last day of the following previous treatments  - Surgery (including exploratory and exploratory open chest): at least 4 weeks  - Continuous thoracic drainage therapy: at least 2 weeks (does not include test puncture or temporary drainage)  - Pleurodesis without antineoplastic agents (BRM such as picibanil): at least 2 weeks  - Antineoplastic drugs: at least 3 weeks |
| 8) ^§^ECOG PS of 0 or 1 or 2 |
| 9) Expected to survive at least 3 months |
| 10) Adequate organ function (liver function, ^¶^PaO_2_, etc.) |
| 11) Written consent from the patient |
| 12) Age: 20 years or older |

^†^EGFR: Epidermal growth factor receptor, ^‡^EGFR-TKI: EGFR-tyrosine kinase inhibitors, ^§^ECOG PS: Eastern Cooperative Oncology Group performance status, ^¶^PaO_2_: Partial pressure of oxygen

**Supplementary Table 2.** Exclusion criteria

| Exclusion criteria |
| --- |
| 1) Already received ^†^EGFR-TKI |
| 2) Interstitial pneumonia or pulmonary fibrosis evident on chest radiograph |
| 3) Heterochronous or simultaneous active multiple or multiple cancers |
| 4) Pleural, ascites, or pericardial effusions requiring drainage |
| 5) The following serious complications  - Poorly controlled angina pectoris, myocardial infarction within 3 months, heart failure  - Diabetes mellitus and hypertension that are difficult to control  - Complicated or suspected severe infectious disease  - Significant or recent gastrointestinal problems with diarrhea as the main symptom  - Other complications that would seriously interfere with treatment (ileus, superior vena cava syndrome, etc.) |
| 6) Inappropriate as a subject of this study by the physician in charge |

^†^EGFR- TKI: Epidermal growth factor receptor-tyrosine kinase inhibitors

**Supplementary Table 3.** EGFR mutations found in pre-treatment biopsies

| ^†^EGFR mutation | Tumor (n = 30) | Liquid (n = 30) |
| --- | --- | --- |
| 19del/T790M | 0 | 0 |
| L858R/T790M | 0 | 0 |
| 19del/ - | 25 (83.3%) | 17 (56.7%) |
| L858R/ - | 5 (16.7%) | 2 (6.7%) |
| Negative | 0 | 11 (36.7%) |
| Unsuccessful | 0 | 0 |
| concordance between tumor and liquid biopsy | 19/30 (63.3%) | |

^†^EGFR: Epidermal growth factor receptor

**Supplementary Table 4.** Results of ^†^EGFR testing among 18 patients who reached ^‡^PD

| No | Stage | Extrathoracic metastasis | Tumor-  EGFR | Pretreatment- liquid biopsy | Intermediary- liquid biopsy | PD- liquid biopsy | PD- re-biopsy | Best response |
| --- | --- | --- | --- | --- | --- | --- | --- | --- |
| 1 | postoperative recurrence | no | 19del | negative | negative | negative | N/A | ^¶^PR |
| 2 | Ⅳ | yes | 19del | 19del | N/A | 19del | 19del | PR |
| 3 | postoperative recurrence | yes | L858R | negative | negative | negative | L858R/T790M | SD |
| 4 | Ⅳ | yes | 19del | 19del | N/A | negative | 19del/T790M | PR |
| 5 | Ⅳ | yes | 19del | 19del | negative | negative | negative | SD |
| 6 | Ⅳ | yes | 19del | 19del | 19del | 19del | N/A | PR |
| 7 | postoperative recurrence | no | L858R | L858R | N/A | L858R | L858R | PD |
| 8 | postoperative recurrence | no | 19del | negative | negative | negative | 19del/T790M | CR |
| 9 | Ⅳ | yes | 19del | 19del | negative | negative | no tumor | PR |
| 10 | Ⅳ | no | 19del | negative | negative | negative | 19del | PR |
| 11 | ⅢB | yes | 19del | 19del | N/A | negative | N/A | ^§^CR |
| 12 | Ⅳ | yes | 19del | 19del | N/A | 19del | 19del | SD |
| 13 | Ⅳ | yes | 19del | 19del | N/A | negative | 19del | SD |
| 14 | Ⅳ | no | 19del | 19del | negative | negative | N/A | SD |
| 15 | Ⅳ | yes | 19del | 19del | negative | 19del/T790M | N/A | PR |
| 16 | Ⅳ | yes | 19del | 19del | negative | 19del/T790M | N/A | PR |
| 17 | Ⅳ | yes | 19del | 19del | 19del | 19del/T790M | N/A | PR |
| 18 | Ⅳ | yes | 19del | 19del | N/A | 19del/T790M | N/A | PR |

^†^EGFR, Epidermal growth factor receptor; ^‡^PD, progressive disease; ^§^CR, complete response; ^¶^PR, partial response; ^††^SD, stable disease; ^‡‡^N/A, not available

**Supplementary Table 5. ^†^**EGFR mutation found by liquid biopsy at intermediate

| EGFR mutation | Liquid (n = 18) |
| --- | --- |
| 19del/T790M | 0 |
| L858R/T790M | 0 |
| 19del/ - | 2 (11.1%) |
| L858R/ - | 0 |
| negative | 16 (88.9%) |
| unsuccessful | 0 |

^†^EGFR: Epidermal growth factor receptor

**Supplementary Table 6.** Safety summary in patients treated with afatinib

|  | （n＝30） |
| --- | --- |
| any ^†^AE, n (%) | 30 (100) |
| any AE grade ≥3, n (%) | 13 (43.3) |
| any AE leading to death, n (%) | 0 (0) |
| any AE leading to discontinuation, n (%) | 6 (20.0) |
| any AE possibly causally related, n (%) | 28 (93.3) |
| any AE possibly causally related Grade ≥3, n (%) | 10 (33.3) |
| any AE possibly causally related leading to death, n (%) | 0 (0) |

^†^AE: Adverse events

**Supplementary Table 7.** All-cause adverse events

|  | (n = 30) | |
| --- | --- | --- |
|  | Any grade | Grade≧3 |
| diarrhea, n (%) | 21 (70.0) | 5 (16.6) |
| rash acneiform, n (%) | 18 (60.0) | 2 (6.6) |
| dry skin, n (%) | 4 (13.3) | 0 (0) |
| pruritus, n (%) | 8 (26.6) | 0 (0) |
| paronychia, n (%) | 15 (50.0) | 2 (6.6) |
| mucositis oral, n (%) | 7 (23.3) | 0 (0) |
| nausea, n (%) | 4 (13.3) | 0 (0) |
| anorexia, n (%) | 5 (16.6) | 2 (6.6) |
| dysgeusia, n (%) | 1 (3.3) | 0 (0) |
| fatigue, n (%) | 2 (6.6) | 1 (3.3) |
| dyspnea, n (%) | 2 (6.6) | 0 (0) |
| pneumonitis, n (%) | 1 (3.3) | 1 (3.3) |
| intracranial hemorrhage, n (%) | 1 (3.3) | 1 (3.3) |
| ^†^ALT・AST increased, n (%) | 1 (3.3) | 1 (3.3) |
| creatinine increased, n (%) | 1 (3.3) | 1 (3.3) |

^†^ALT AST: Aspartate aminotransferase (AST) and alanine aminotransferase (ALT)
